# Effect of saw palmetto extract on PI3K cell signaling transduction in human glioma

**DOI:** 10.3892/etm.2014.1756

**Published:** 2014-06-04

**Authors:** YANG YANG, LV HUI, CHE YUQIN, LI JIE, HOU SHUAI, ZHOU TIEZHU, WANG WEI

**Affiliations:** Department of Neurology, The Fourth Affiliated Hospital of China Medical University, Shenyang, Liaoning 110032, P.R. China

**Keywords:** saw palmetto extract, human glioma, p53, phosphatidylinositol 3-kinase/Akt

## Abstract

Saw palmetto extract can induce the apoptosis of prostate cancer cells. The aim of the present study was to investigate the effect of saw palmetto extract on the phosphatidylinositol 3-kinase (PI3K)/Akt signaling transduction pathway in human glioma U87 and U251 cell lines. Suspensions of U87 and U251 cells in a logarithmic growth phase were seeded into six-well plates at a density of 10^4^ cells/well. In the experimental group, 1 μl/ml saw palmetto extract was added, while the control group was cultured without a drug for 24 h. The expression levels of PI3K, B-cell lymphoma-extra large (Bcl-xL) and p53 were evaluated through western blot analysis. In the experimental group, the U87 and U251 cells exhibited a lower expression level of PI3K protein as compared with the control group (t=6.849; P<0.001). In addition, the two cell lines had a higher expression level of p53 protein in the experimental group as compared with the control group (t=40.810; P<0.001). Protein expression levels of Bcl-xL decreased significantly in the experimental group as compared with the control group (t=19.640; P=0.000). Therefore, saw palmetto extract induces glioma cell growth arrest and apoptosis via decreasing PI3K/Akt signal transduction.

## Introduction

Human gliomas originate from neural stromal cells, including glial, ependyma, choroid plexus epithelial and neural parenchymal cells. The incidence rate in adults is ~6/100,000 and the five-year survival rate is between 20 and 30%. Gliomas account for 35.26–60.96% of central nervous system tumors (average, 44.69%) (1). The tumors exhibit infiltrating growth and have no evident boundary with the normal brain tissue, thus, it is difficult to completely resect gliomas via surgery. In addition, gliomas are not sensitive to radiotherapy or chemotherapy, and have one of the worst prognoses for systemic tumors (2). Previous studies have shown that the phosphatidylinositol 3-kinase (PI3K)/Akt signaling transduction pathway plays a central role in the maintenance of malignant glioma invasion (3). The activation of this pathway can promote the proliferation of tumor cells and inhibit the apoptosis of tumor cells. The dependence on PI3K/Akt signaling transduction makes this pathway an attractive target for the treatment of gliomas (4). In previous research of our group, saw palmetto extract was shown to markedly inhibit the proliferation of human glioma cells, and the underlying mechanism may be associated with the inhibition of signal transducer and activator of transcription 3 phosphorylation. Additionally, previous studies have demonstrated that saw palmetto extract can induce the apoptosis of prostate cancer cells (5). In previous research, the effect of saw palmetto extract on human glioma U87 and U251 cells was investigated *in vitro*. The results revealed that saw palmetto extract markedly inhibited the proliferation of human glioma cells and the underlying mechanism may be associated with the inhibition of signal transducer and activator of transcription 3 (STAT3) phosphorylation. Therefore, the aim of the present study was to investigate the effect of saw palmetto extract on the PI3K/Akt signaling transduction pathway in human glioma U87 and U251 cell lines.

## Materials and methods

### Human glioma cell lines

U87 and U251 cell lines were purchased from Beijing Dingguochangsheng Biotech Co., Ltd. (Beijing, China).

### Experimental apparatus

A T25 cell culture flask and 6-well cell culture plates were purchased from Corning Inc. (Corning, NY, USA). A 3K30 model centrifuge (Sigma-Aldrich, St.Louis, MO, USA) and a BX51 model microscope (Olympus, Tokyo, Japan) were used, as well as a Chemilmager 5500 vertical imager (Alpha Innotech, San Leandro, CA, USA), Protean IIXi + PowerPac 3000, Bio-Rad, Herculaes, CA, USA) electrophoresis device and a blood cell counting plate (model 3100; Hausser Scientific, Horsham, PA, USA).

### Reagents and drug

Saw palmetto extract was purchased from Yongyuan Bio-technology, Co., Ltd. (Xi’an, China). Rabbit anti-B-cell lymphoma-extra large (Bcl-xL), anti-p53, anti-PI3K and anti-β-actin antibodies were purchased from Bioss, Inc. (Wuhan, China). An enhanced chemiluminescence (ECL) kit was obtained from the Beyotime Institute of Biotechnology (Shanghai, China).

### Cell culture

Human brain glioma cell lines, U87 and U251, were grown in a 25-cm^2^ cell culture bottle containing Dulbecco’s modified Eagle’s medium, supplemented with 10% fetal bovine serum, 100 IU/ml penicillin and 100 μg/ml streptomycin, at 37°C and 5% carbon dioxide. Cell growth was observed under an inverted microscope and the medium was replaced every two days.

### Cell count

A counting plate and cover glass was cleaned with 95% alcohol. Take ~1 μl of cell suspension and drip on the blood counting chamber to count the cell concentration. Under a microscope at ×10 magnification, the cell number was counted with four angles in the grid on the plate. The procedure was performed in triplicate. The cell number was calculated as follows: Cell number (/ml) = (total cell number of the four angles/4) × 10^4^ × dilution.

### Western blot analysis

U87 and U251 cell suspensions in a logarithmic growth phase were seeded into six-well plates at a density of 10^4^ cells/well. In the experimental group, 1 μl/ml saw palmetto extract was added, while no drug was added to the control group. The suspensions were cultured for 24 h. Next, the supernatant was discarded and the plates were washed twice with phosphate-buffered saline (PBS) at 4°C. Radioimmunoprecipitation assay buffer (RIPA) cell lysate with phenylmethylsulfonyl fluoride (PMSF) was added to each well (~80 μl), which was followed by gentle shaking for 30 min on ice and repeated pipetting and scraping of the bottom of the well. The lysate was fully removed and placed into a 1.5-ml centrifuge tube. The samples were then centrifuged for 25 min at 4°C under 14,000 × g. The supernatant was then collected and the protein concentration was measured using the Bradford method.

Proteins were separated by sodium dodecyl sulfate-polyacrylamide gel electrophoresis, using a 6–15% acrylamide resolving gel, and transferred to polyvinylidene fluoride membranes. The membranes were blocked with Tris-buffered saline-Tween-20 (0.1% Tween-20) containing 5% milk for 60 min at room temperature, which was followed by incubation with primary antibodies at 4°C overnight. Rabbit anti-Bcl-xL (1:200), anti-p53 (1:200), anti-PI3K (1:200) and anti-β-actin antibodies (1:5,000) were used to detect the target proteins. The blots were then probed with horseradish peroxidase-conjugated anti-rabbit IgG (1:5,000; Beyotime Institute of Biotechnology). According to the manufacturer’s instructions of the ECL kit, the same volume of solution A and B were mixed and reacted for 1 min.

Densitometry was performed using the Quantity One software (Bio-Rad Laboratories, Hercules, CA, USA).

### Statistical analysis

All data were analyzed using SPSS 15.0 statistical software (SPSS, Inc., Chicago, IL, USA). Data are expressed as the mean ± standard deviation. Comparisons between the two groups were conducted using the t-test, where P<0.05 was considered to indicate a statistically significant difference.

## Results

### Effect of saw palmetto extract on the protein expression levels of PI3K in U87 glioma cells

**Figure 1:**
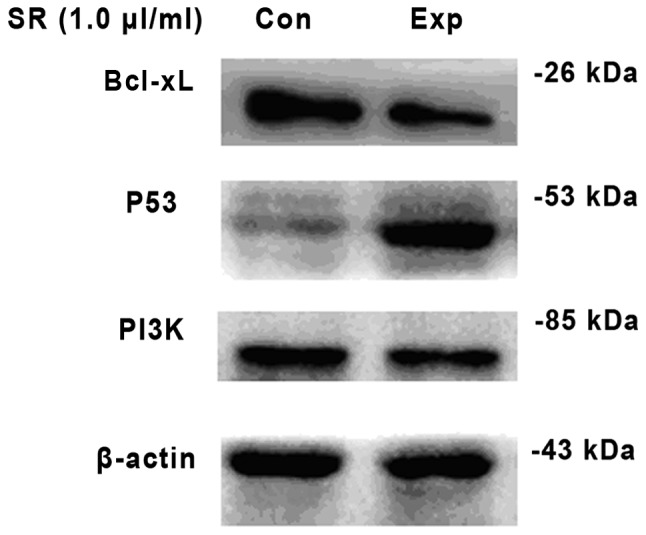
Protein expression levels of Bcl-xL, p53 and PI3K in glioma U87 cells were measured by western blot analysis. β-actin was used as an internal control. The left of the figure indicates the control group, while the right indicates the experimental group. SR (1.0 μl/ml) indicates that the experimental group was treated with saw palmetto extract at a concentration of 1.0 μl/ml. PI3K, phosphatidylinositol 3-kinase; Bcl-xL, B-cell lymphoma-extra large.

Following treatment with saw palmetto extract, the protein expression level of PI3K had significantly decreased when compared with the control group in U87 glioma cells (t=13.959; P=0.000). In addition, p53 protein expression increased significantly as compared with the untreated U87 cells (t=12.440; P= 0.000), and U87 cells pretreated with saw palmetto extract exhibited significantly decreased Bcl-xL protein expression as compared with the control (t=15.107; P<0.001; Table I; Fig. 1).

### Effect of saw palmetto extract on the protein expression levels of PI3K in U251 glioma cells

**Figure 2:**
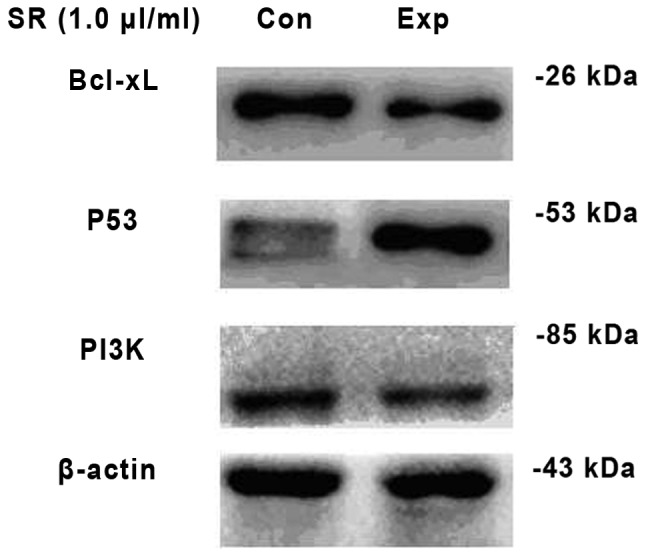
Protein expression levels of Bcl-xL, p53 and PI3K in glioma U251 cells were measured by western blot analysis. β-actin was used as an internal control. The left of the figure indicates the control group, while the right indicates the experimental group. SR (1.0 μl/ml) indicates that the experimental group was treated with saw palmetto extract at a concentration of 1.0 μl/ml. PI3K, phosphatidylinositol 3-kinase; Bcl-xL, B-cell lymphoma-extra large.

Following treatment with saw palmetto extract, U251 cells exhibited a lower expression level of PI3K protein as compared with the control group (t=6.849; P<0.001). However, the U251 cells exhibited a higher expression level of p53 protein as compared with the untreated group (t=40.810; P<0.001). In addition, the Bcl-xL protein expression decreased significantly in the experimental group as compared with the untreated control (t=19.640; P<0.001; Table II; Fig. 2).

## Discussion

Gliomas are the most common primary tumors, accounting for ~46% of intracranial tumors and ~2% of adult tumors. The majority of gliomas are characterized by invasive growth and are difficult to completely resect via surgery. In addition, chemotherapy and radiotherapy exhibit poor therapeutic effects. In previous years, studies have focused on chemical compounds derived from plants that possess pharmacological activity in antitumor therapy (6,7). A previous study demonstrated that a number of chemical compounds in herbaceous plant sources can inhibit the proliferation of tumor cells and induce apoptosis through altering the tumor metabolism, inhibiting the cell cycle and activating the immune response (8).

The effective components of saw palmetto extract, as matured dry fruit of saw palm, are fatty acids. Previously, a foreign study reported that saw palmetto extract had inhibitory effects on the proliferation and induction of apoptosis in prostate cancer cells (9). The main pharmacological effects of saw palmetto extract are as follows: i) Non-competitive inhibition of 5-α reductase activity and inhibition of the conversion of testosterone to dihydrotestosterone (DHT); ii) inhibition of the binding of DHT to the androgen receptor; and iii) anti-inflammatory effects. However, the effect on brain gliomas remains to be fully understood. In early *in vitro* experiments, the effect of saw palmetto extract on human glioma U87 and U251 cells was studied. The results revealed that saw palmetto extract markedly inhibited the proliferation of human glioma cells. The mechanism may be associated with the inhibition of STAT3 phosphorylation. Previous studies have hypothesized that the STAT3 and PI3K/Akt signaling transduction pathway has an important role in tumor cells (10,11,12). In the present study, the influence of saw palmetto extract on human glioma U87 and U251 cell lines was further discussed with regard to PI3K/Akt signaling transduction.

An increasing number of studies have demonstrated that the PI3K/Akt signaling transduction pathway plays an important role in the occurrence and development of malignant gliomas (13–15). The PI3K/Akt signaling pathway is not only important in cell proliferation and apoptosis, but also in tumor growth and the response to chemotherapy (16). Activation of Akt can directly phosphorylate a number of transcription factors, which regulates the transcription factor, inhibits the expression of apoptosis-related genes and enhances the expression of antiapoptotic genes, including the Bcl-2 family, p53 and the FKHR forkhead transcription factor. A previous study demonstrated that proto-oncogenes and anti-oncogenes are involved in the regulation of apoptosis (17). Bcl-2 is one of the original cancer genes that was identified to be associated with apoptosis (18). Bcl-2 is capable of encoding 1G5M (26 kDa) and 1GO/JH (22 kDa) proteins that exist in the outer mitochondrial membrane, nuclear membrane and endoplasmic reticulum, and the Bcl-2 encoded protein is involved in maintaining the integrity of the mitochondrial membrane. According to the variety of structures and functions, the Bcl-2 family can be divided into two categories: Inhibition of apoptosis family members (including Bcl-2 and Bcl-xL) and promotion of apoptosis family members (including Bcl, XS, BAX, BAK, BID and BAD). Previously, Bcl-xL, as a member of the Bcl-2 family, was shown to be widely expressed in human tissues and could inhibit cell apoptosis. Bcl-xL can combine with proapoptotic proteins (primarily BAX and BAK) to form a heterologous dipolymer, which improves the survival rate of cells via stabilizing the mitochondrial membrane potential, maintaining the mitochondrial outer membrane integrity and preventing the release of cytochrome *c* and apoptosis inhibition factor. Under an apoptosis signal, Bcl-xL can release from BAX, resulting in cell apoptosis through altering the permeability of the mitochondrial outer membrane and releasing cytochrome *c* and other proapoptotic material. Lower protein expression of Bcl-xL has been identified in normal brain tissue, while higher levels have been identified in gastric, esophageal and gallbladder cancers, among other tumor tissues. Bcl-xL is hypothesized to be closely associated with the occurrence and development of malignant tumors (18). According to the results of the present study, the expression level of PI3K and Bcl-xL decreased significantly following treatment with saw palmetto extract in glioma cells. Thus, the results indicate that saw palmetto extract downregulates the PI3K/Akt signaling transduction pathway and inhibits the expression of Bcl-xL, known as the downstream signaling factor of the PI3K/Akt signaling transduction pathway (19).

p53 is an important protein that mediates DNA damage-associated apoptosis. The levels and functions of p53 can be decreased by MDM2 ubiquitin ligase (20). As the downstream signaling molecule of the PI3K/Akt pathway, MDM2 ubiquitin ligase is a negatively regulated protein of p53. Akt is able to combine with MDM2 and phosphorylate the Ser 66 and Ser 88 sites in MDM2. Upregulated activity of ubiquitin ligase promotes p53 inactivation or degradation. The normal p53 gene consists of 11 exons and encodes a 53 kDa nuclear phosphoprotein, which is a tumor suppressor gene located on the short arm of chromosome 17 (21). p53 is associated with multiple cellular processes, including gene transcription, DNA repair, cell cycle, genome stability, chromosome separation, cell senescence and programmed cell death (22). The p53 gene is one of the most frequently mutated genes in human cancers, accounting for one third of gliomas. The mutant p53 gene not only loses its tumor suppressor capability, but also promotes malignant transformation. There a are a number of mechanisms underlying p53 gene transfer between tumor suppressor and oncogene and the regulation of cell proliferation. Firstly, wild type p53 is a negative regulator of cell growth, however, the inactivation of the p53 gene induced by gene loss or mutation results in the growth advantage of transformed cells and tumor cells. Secondly, mutated p53 protein can bind to the subunit of wild-type p53 to form oligomeric complexes, which interfere with the function of the wild-type subunits. Finally, N-terminal transcriptional activators of mutated p53 protein can regulate the expression of c-fos, c-myc and other cancer genes associated with the proliferation and differentiation of cells (23). The results of the present study indicate that the protein expression level of p53 in glioma cells treated with saw palmetto extract was significantly higher than in the control group. Thus, the expression of p53 increased due to the lower expression levels of the p53 inhibitor, MDM2, following the inhibition of the PI3K/Akt signaling transduction pathway (24).

The etiology of gliomas is yet to be fully understood. Investigating the occurrence and development of tumor cells that have resulted from the abnormal activation of PI3K signaling transduction is important. Saw palmetto extract can induce glioma cell growth arrest and apoptosis through decreasing PI3K/Akt signaling transduction. Therefore, the PI3K signaling transduction pathway takes part in the proliferation of glioma. We speculate that interferance with the PI3K signaling pathway maybe beneficial to the treatment of glioma in the future.

## Figures and Tables

**Table I:** Comparison in the OD of PI3K, Bcl-xL and p53 in U87 cells between the experimental and control groups (mean ± SD; n=6).

Groups	PI3K	Bcl-xL	p53
Control	0.58±0.076	1.08±0.114	0.23±0.045
Experimental	0.36±0.041^a^	0.34±0.034^a^	1.25±0.052^a^

a P<0.05, vs. control.

OD, optical density; PI3K, phosphatidylinositol 3-kinase; Bcl-xL, B-cell lymphoma-extra large.

**Table II:** Comparison in the OD of PI3K, Bcl-xL and p53 in U251 cells between the experimental and control groups (mean ± SD; n=6).

Groups	PI3K	Bcl-xL	p53
Control	0.96±0.166	0.92±0.059	0.25±0.039
Experimental	0.39±0.119^a^	0.33±0.043^a^	1.30±0.049^a^

a P*<*0.05, vs. control.

OD, optical density; PI3K, phosphatidylinositol 3-kinase; Bcl-xL, B-cell lymphoma-extra large.
